# Analyzing state dyslexia legislation through the lens of oral language: an exploratory study

**DOI:** 10.1007/s11881-026-00380-3

**Published:** 2026-07-02

**Authors:** Wendy C. Georgan

**Affiliations:** Division of Speech-Language Pathology, New York Medical College, 30 Plaza W, Valhalla, NY 10595 USA

**Keywords:** Dyslexia legislation, Oral language, Screening, Language disorder

## Abstract

Recent advocacy efforts have resulted in 49 out of 50 U.S. states passing some form of dyslexia legislation. This legislation may serve as a starting point for educators, clinicians, and parents to advocate for universal oral language screening as well as dyslexia screening. The purpose of this exploratory study is to investigate the degree to which oral language skills are already included in existing policies. We used deductive and inductive coding procedures to identify key words related to oral language skills. We then performed document analysis on 156 legislative documents related to dyslexia to describe the following: (a) to what extent states include “language disorder” or other related terms in their descriptions of disorders encompassed by the legislation; (b) to what extent legislation includes keywords related to oral language; and (c) how language keywords are distributed across screening, intervention, preservice preparation, and professional development requirements. Two states include “language disorder” in the scope of dyslexia legislation. An additional eight states have legislation with terms that could encompass a language disorder. Out of the 49 states with dyslexia legislation, 29 include at least one language-related keyword. The most common language keywords found in such legislation were “comprehension” and “vocabulary.” Keywords were mentioned across screening, intervention, preservice preparation, and professional development requirements. In many states, existing legislation lays the groundwork for implementing universal oral language skill screening. Further legislation dedicated to universal oral language screening is also necessary to ensure children with language disorders are identified early.

## Introduction

The ability to read is perhaps one of the most pervasive predictors of life outcomes. Children who struggle with reading and writing are more likely to drop out of high school (Kutner et al., 2007; Lloyd, 1978) or end up in the juvenile justice system (Ritchie et al., 2002; Keilitz & Dunivant, 1986). Adults with low health literacy tend to have more hospitalizations and higher mortality rates (Berkman et al., 2011). Unfortunately, according to the 2024 U.S. National Assessment of Education Progress (NAEP) report, 40% of fourth-grade students did not meet basic reading proficiency (National Center for Education Statistics, 2024). Students who struggle to read may have difficulties with word-level decoding, language comprehension (e.g., oral language skills), or both (Gough & Tunmer, 1986).

Children who struggle with word-level decoding may be at risk for dyslexia, while children who have difficulties understanding and using oral language may be at risk for language disorders, including Developmental Language Disorder (DLD). Additionally, there is a high rate of comorbidity between dyslexia and DLD, such that around 50% of children with DLD also have dyslexia (Snowling et al., 2019). Over the past few decades, 49 out of 50 states in the United States have passed legislation related to identifying children with dyslexia through the public education system. However, children with DLD are woefully under-identified (Hendricks et al., 2019; see also Tomblin et al., 1997), and schools do not systematically screen for oral language skills (Adlof & Hogan, 2019). This exploratory review was motivated by the disparity in legislative action that has been taken to screen for children with dyslexia versus children with DLD. In this paper, we explore current dyslexia legislation to identify areas where existing legislation could be interpreted to include language screening, as well as to highlight the need for more specific legislation dedicated to identifying children with DLD.

### Dyslexia and DLD defined: the simple view of reading

The simple view of reading (Gough & Tunmer, 1986) is a widely used framework that breaks down reading comprehension into two main components: (1) word recognition (e.g., word-level decoding) and (2) language comprehension. This model suggests that deficits in either one of these components will result in poor reading comprehension, and that the relative contributions of each component change over time. In particular, previous studies have found that word-level decoding skills are more predictive of reading ability in early elementary grades, while older children show a stronger correlation between oral language skills and reading comprehension (Catts et al., 2005). In addition, children who have weak oral language skills but good decoding skills are at risk of being unidentified during early grades, when their relative strengths in decoding may mask language deficits due to relatively unimpaired reading comprehension (Catts et al., 2012).

Difficulties with word-level decoding are encompassed in the definition of dyslexia. At the time of this study, the most current definition used by the International Dyslexia Association (IDA) read as follows^1^:

Dyslexia is a specific learning disability that is neurobiological in origin. It is characterized by difficulties with accurate and/or fluent word recognition and by poor spelling and decoding abilities. These difficulties typically result from a deficit in the phonological component of language that is often unexpected in relation to other cognitive abilities and the provision of effective classroom instruction. Secondary consequences may include problems in reading comprehension and reduced reading experience that can impede growth of vocabulary and background knowledge (IDA, 2002).

This definition explicitly identifies secondary consequences related to vocabulary and reading comprehension, of which language comprehension is a key component. Research has shown that word-level reading difficulties can lead to weaker language skills in later grades, as much language information is gained through “reading to learn” (Catts et al., 1999).

In contrast, difficulties with oral language beyond the secondary consequences related to dyslexia fall under the definition of a language disorder, including developmental language disorder (DLD). DLD is a lifelong neurodevelopmental disorder that impacts one’s ability to understand and use language in the absence of brain damage, hearing impairment, or intellectual disability (McGregor et al., 2020). Its presentation is heterogenous, and people with DLD can have difficulties in word learning and vocabulary, morphosyntactic skills, and discourse-level language (Lancaster & Camarata, 2019). Children with DLD are at greater risk for having reading difficulties as a result of poor oral language skills (Catts et al., 2002; Snowling et al., 2000).

### Dyslexia legislation: a potential avenue for language screening

In recent years, advocacy groups such as Decoding Dyslexia have pushed for state-level legislation that mandates universal screening and intervention to identify students with dyslexia (Gearin et al., 2018). As of April 2025, 49 out of 50 states in the U.S. have passed dyslexia-related legislation, and the remaining state of Hawaii has introduced a senate bill that would require schools to administer dyslexia screening beginning in the 2026–27 school year (Hawaii State Legislature, 2025). The scope of these laws varies significantly from state to state. Current state laws have some combination of screening requirements related to dyslexia, requirements for intervention for students identified through screening, mandated dyslexia training for preservice preparation (i.e. teacher training programs), and mandated dyslexia training for in-service providers (i.e. professional development) (National Center on Improving Literacy, 2026).

Additionally, at the federal level, the Individuals with Disabilities Education Act (IDEA) requires that students whose disabilities affect their educational performance be provided special education services under certain eligibility categories (IDEA, 2004). One of these categories is “Specific Learning Disability,” for which the legal definition includes “dyslexia” as one of the conditions that falls into this category. Furthermore, in 2015, the Office of Special Education and Rehabilitative Services issued a Dear Colleague letter clarifying that “there is nothing in the IDEA that would prohibit the use of the terms dyslexia, dyscalculia, and dysgraphia in IDEA evaluation, eligibility determinations, or IEP documents” (Yudin, 2015). This letter was issued in response to advocacy from parent groups, state organizations, and national disability organizations, who stated that schools were often reluctant to use the term “dyslexia” in their evaluative processes.

Though there is heterogeneity in how these state laws are written and implemented (Gearin et al., 2021; Odegard et al., 2026), there are several key aspects of dyslexia legislation and how the term “dyslexia” is defined that indicate potential for existing dyslexia legislation to serve as a jumping off point for universal language screening. For one, recent reviews of dyslexia legislation have found that most states recognize the IDA definition of dyslexia (Gearin et al., 2021), which includes mention of “vocabulary” and “comprehension” as secondary consequences of word-reading difficulties. Although these language skills are not primarily implicated in dyslexia, some state legislation may include requirements for screening for vocabulary and comprehension, as well as phonological awareness skills, in order to assess these related skills mentioned in the IDA definition.

Additionally, dyslexia has traditionally been defined as a language-based learning disability (Adlof & Hogan, 2018; Lyon et al., 2003), in which phonology is usually identified as the primary component of language that is affected by dyslexia. For states that view dyslexia through a language-based learning disability framework, it is possible that the language of their dyslexia-related legislation may be intentionally broad or vague, such that the legislation could apply to both dyslexia and language disorders. For example, a cursory review of the Massachusetts dyslexia screening law reveals that screening is mandated for a “neurological learning disability including, but not limited to, dyslexia” (Massachusetts Session Laws, 2018). As such, this legislation could be interpreted to include screening for language disorders that co-exist with a learning disability (e.g., a language-based learning disability). In addition, a recent viewpoint has emerged that DLD should be thought of as a language-based learning disability that meets the definitional criteria of “Specific Learning Disability” as stated in the IDEA (Kirkley, 2026).

### Study purpose

The purpose of the current study is to investigate to what degree oral language skills are mentioned and included in existing dyslexia legislation. Language screening in early grades is crucial for identifying students with language disorders who have historically been identified late or not at all (Adlof & Hogan, 2019; Weiler et al., 2018). Children with language disorders often struggle with reading comprehension, and current reading and dyslexia legislation may be a starting point for advocating for and implementing language screening in addition to dyslexia screening in schools. Analyzing current legislation through document analysis is an important first step to identify to what extent language skills are already incorporated into screening, intervention, and training requirements.

## Methods

We applied document analysis methods to a total of 156 legislative documents from 49 states. Document analysis is a useful qualitative research method that lends itself to the investigation of a single phenomenon (Bowen, 2009) and lends itself well to the type of exploratory investigation which is the aim of this study. Moreover, document analysis has been used as a method of analyzing dyslexia legislation specifically in several previous studies (Gearin et al., 2018, 2021).

### Data

To locate documents, we performed online searches across several websites during the period of May to July, 2022. We searched for legislation related to reading and dyslexia using Google; the National Center on Improving Literacy’s “State of Dyslexia”; Dyslegia.com; the National Conference for State Legislators bill tracking database; DyslexiaIDA.org; and state-specific advocacy sites, such as Decoding Dyslexia. We identified 156 legislative documents in total, which includes bills, statutes, administrative codes, acts, and resolutions from all U.S. states excluding Hawaii, which had not passed dyslexia-related legislation at the time of this search. It is important to note that only legislative documents were included in this analysis. In addition to legislation, many states also publish handbooks and resource guides relaying information about how to implement dyslexia screening and intervention in more detail (Gearin et al., 2018; see Brown-Chidsey et al., 2024 for a review of states’ handbooks). These documents likely contain much more detail about screening and intervention targets, and potentially include references to language-related skills. For the purposes of this exploratory study, we focused our search on legislation and did not include these additional documents in our analysis.

### Coding and document analysis

Documents for this study were analyzed in three phases. In the first phase, we determined if language disorders fell into the scope of state dyslexia legislation through a narrow and broad search. For the narrow search, all legislative documents were searched for the specific term “language disorder” to determine if this terminology appeared in the scope of state dyslexia laws. For the broad search, documents were read to see if they contained more vague references to disorders other than dyslexia that would fall into the purview of the legislation. These vague references were noted if they were determined to potentially encompass language disorder. For example, if the scope of a legislative document was written as “dyslexia and other language-based learning disabilities,” we identified this document as having a vague reference that could be interpreted to include language disorders.

In the second phase, legislative documents were coded through a combination of deductive and inductive coding procedures. Through an iterative process of generating keywords and phrases that are pertinent to oral language skills, and using inductive coding methods to extract relevant keywords from a subset of documents, six language-related codes were generated: (1) “receptive language”; (2) “expressive language”; (3) “language development”; (4) “oral language” or “spoken language”; (5) “comprehension”; and (6) “vocabulary.” The purpose of this coding exercise was to locate language-related codes in sections of legislation that mandated screening, intervention, or training requirements. Notably, the final two codes appear in the IDA definition of dyslexia, which is written out verbatim in many of the legislative documents included in this analysis. As such, in order to capture mentions of these codes in contexts of interest to this study, appearances of “vocabulary” and “comprehension” in the context of defining dyslexia were not counted. Additionally, the code “language development” was not counted if it appeared in the phrase “English language development,” as this phrase typically arose in regulations related to the instruction of students who are English language learners. Finally, codes were only counted if they appeared outside the context of defining terminology. For example, many legislative documents begin with a glossary of terms referenced in the document, so mentions of codes in this glossary were not counted in the coding process.

We coded all legislative documents using the six language-related codes and recorded in a spreadsheet whether or not they were present in a document, as well as the number of instances that a code appeared in a document. Then, in order to be able to compare legislation across states, we collapsed coded entries by state in a separate spreadsheet and noted the number of unique codes that appeared in legislation from a given state. For example, we collected six legislative documents from the state of Connecticut and coded each document using the six language codes we generated, noting the number of occurrences of each code in each document. We found one document that contained the codes “vocabulary” and “comprehension,” and a second document that mentioned “oral language.” Then, to characterize Connecticut’s dyslexia legislation as a whole, we recorded in the second spreadsheet that Connecticut’s legislation mentioned three of the six codes across all documents from that state.

Finally, in the third phase of analysis, we recorded the contexts in which language-related codes appeared in each legislative document. We defined contexts based on previous work in characterizing dyslexia legislation through qualitative analysis (Gearin et al., 2018, 2021) and determined four possible contexts in which codes could appear: (1) screening or assessment approaches; (2) intervention approaches; (3) preservice preparation, and (4) professional development. We then reviewed each legislative document to determine the contexts in which language-related codes appeared and recorded them in a spreadsheet. These entries were also collapsed by state to characterize all of the contexts in which codes appeared in a given state’s dyslexia legislation.

## Results

### Scope of dyslexia legislation

Through the first phase of analysis, we found that two states had legislation that contained specific reference to “language disorder” in their description of the scope of legislation: Georgia and Oklahoma. In Georgia, “language disorder” is mentioned in the scope of a law pertaining to the standards for a professional dyslexia endorsement, such that educators seeking this certification must be trained in “recognizing and responding to students with characteristics of dyslexia and **language disorders**, such as difficulty with expressive or receptive language” (Georgia General Assembly Legislation, 2019). In Oklahoma, “language disorder” is mentioned in the scope of a screening law which requires the Oklahoma State Board of Education to “develop policies for dyslexia screening” that “include… the definition and characteristics of dyslexia and related **language disorders**” (Oklahoma State Legislature, 2020). When we applied a broader search to include vague references that could encompass language disorders, we identified eight states that used vague terminology. Table 1 summarizes these findings.

**Table 1:** States with legislation that uses vague references to disorders other than dyslexia and which could encompass a language disorder

State	Reference phrase
California	*“developmental aphasia”*
Colorado	*“literacy challenge”*
Connecticut	*“reading-related learning disabilities”*
Florida	*“other learning disorders”*
Massachusetts	*“neurological learning disability”*
Missouri	*“other language difficulties”*
Virginia	*“language-based learning difficulties”*
West Virginia	*“developmental aphasia”*

### Language-related codes in dyslexia legislation

**Fig. 1:**
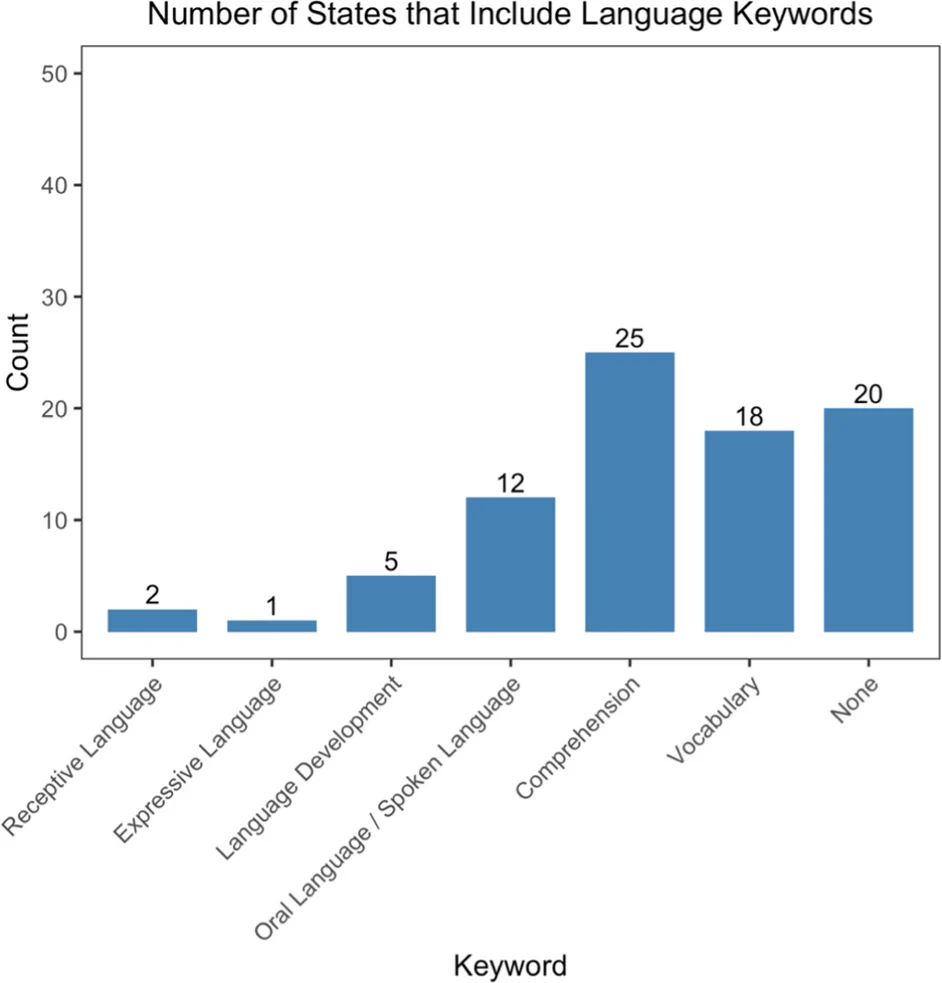
Number of states that included each of the six language codes defined in the study

Overall, we found that language-related codes appeared in the legislation of 29 states. Table 2 provides a summary of which codes appeared in which states. The most common keywords were “comprehension,” which appeared in legislation from 25 states, and “vocabulary,” which appeared in legislation from 18 states. There were 20 states whose legislation did not include any of the language-related codes. The number of instances of each code across all legislative documents included in this study is depicted in Fig. 1.

**Table 2:** Language-related codes identified in state dyslexia legislation

State	Receptive Language	Expressive Language	Language Develop-ment	Oral Language	Compre-hension	Vocab-ulary	Total Unique Codes
Alabama	0	0	1	1	1	1	4
Alaska	0	0	0	0	0	0	0
Arizona	0	0	0	0	1	1	2
Arkansas	0	0	0	0	1	0	1
California	0	0	0	1	1	0	2
Colorado	0	0	0	0	1	1	2
Connecticut	0	0	0	1	1	1	3
Delaware	0	0	0	0	0	0	0
Florida	0	0	1	1	1	1	4
Georgia	1	1	0	0	0	0	2
Idaho	0	0	0	0	1	0	1
Illinois	0	0	0	0	0	0	0
Indiana	0	0	0	0	1	0	1
Iowa	0	0	0	0	1	1	2
Kansas	0	0	0	0	0	0	0
Kentucky	0	0	0	0	1	1	2
Louisiana	1	0	0	1	1	1	4
Maine	0	0	0	0	0	0	0
Maryland	0	0	0	0	1	1	2
Massachusetts	0	0	0	0	0	0	0
Michigan	0	0	0	1	1	1	3
Minnesota	0	0	1	1	1	1	4
Mississippi	0	0	0	0	1	0	1
Missouri	0	0	1	1	1	1	4
Montana	0	0	0	0	0	0	0
Nebraska	0	0	0	0	1	1	2
Nevada	0	0	0	0	1	0	1
New Hampshire	0	0	0	1	0	0	1
New Jersey	0	0	0	1	1	1	3
New Mexico	0	0	0	1	0	0	1
New York	0	0	0	0	0	0	0
North Carolina	0	0	0	0	0	0	0
North Dakota	0	0	0	0	0	0	0
Ohio	0	0	0	0	0	0	0
Oklahoma	0	0	1	0	0	0	1
Oregon	0	0	0	0	0	0	0
Pennsylvania	0	0	0	0	0	0	0
Rhode Island	0	0	0	0	0	0	0
South Carolina	0	0	0	0	1	1	2
South Dakota	0	0	0	0	0	0	0
Tennessee	0	0	0	0	1	1	2
Texas	0	0	0	0	1	1	2
Utah	0	0	0	0	0	0	0
Vermont	0	0	0	0	0	0	0
Virginia	0	0	0	1	1	1	3
Washington	0	0	0	0	0	0	0
West Virginia	0	0	0	0	0	0	0
Wisconsin	0	0	0	0	0	0	0
Wyoming	0	0	0	0	1	0	1

**Fig. 2:**
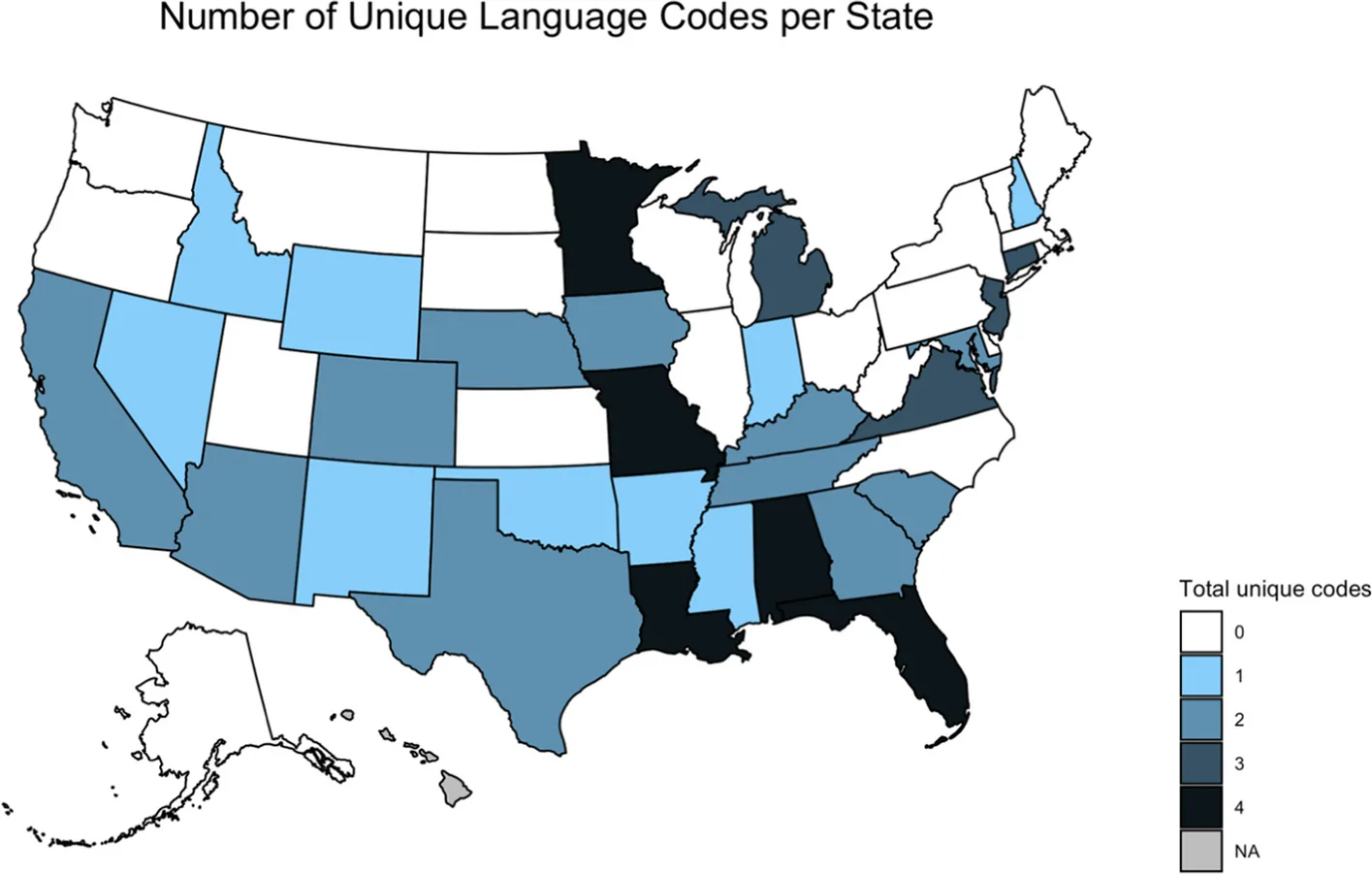
Number of unique language codes (out of 6) included in dyslexia legislation for each of the 49 relevant states (excluding Hawaii due to lack of related legislation). For example, if there were three mentions of “vocabulary” and two mentions of “comprehension”, the total unique codes mentioned would be two

When looking state by state, we found a total of 20 states that did not include any language keywords; 9 states that included one keyword; 11 states that included two keywords; 4 states that included three keywords; and 5 states that included four keywords. Four was the maximum number of unique keywords that were found in the legislation from a single state. These results are depicted in the map in Fig. 2.

### Contexts of language code appearances

**Fig. 3:**
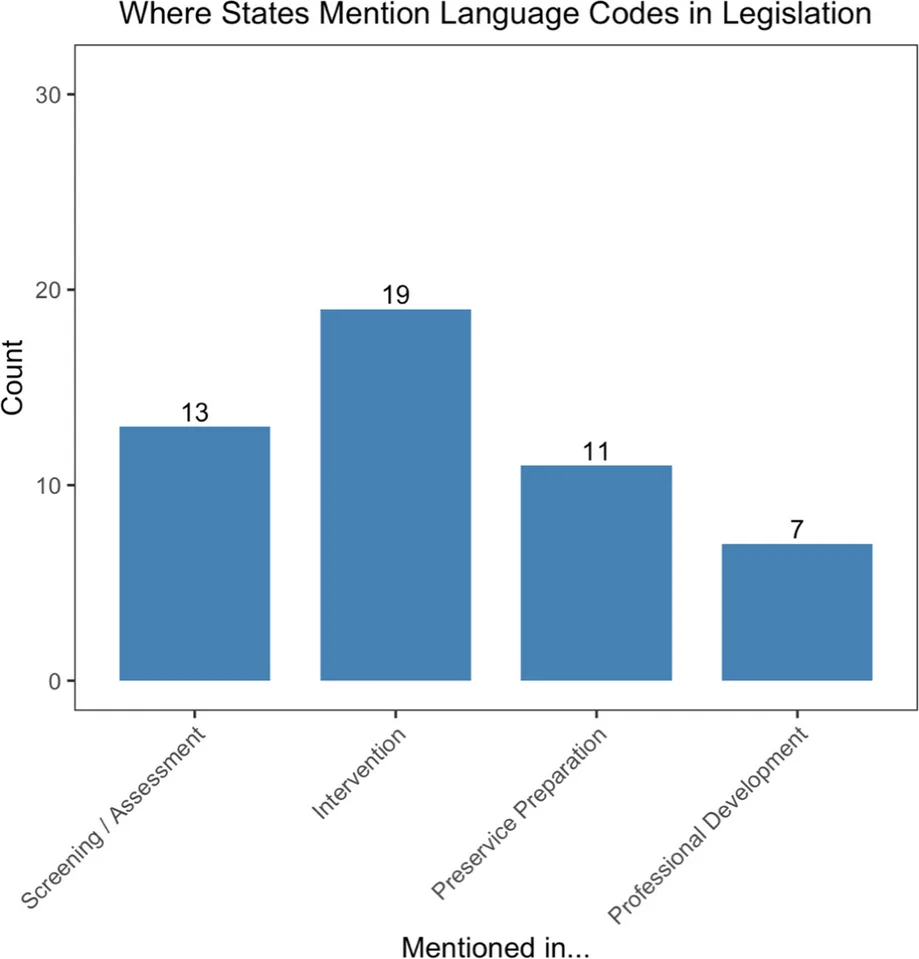
Number of states that use language codes in different contexts of legislation

We further investigated the 29 states that had legislation that mentioned at least one language code to characterize the context in which these codes were mentioned. Detailed results can be found in Table 3. Most states had language codes appearing in sections related to intervention (19 states). Overall, language codes were found across all four contexts defined in this study (screening/assessment, intervention, preservice preparation, and professional development). These results are summarized in Fig. 3.

**Table 3:** Contexts in which language codes appeared in state dyslexia legislation

State	Screening/Assessment	Intervention	Preservice Preparation	Professional Development
Alabama	Y	Y	Y	Y
Arizona	N	N	Y	N
Arkansas	N	Y	N	N
California	N	N	Y	N
Colorado	Y	Y	N	N
Connecticut	Y	N	N	N
Florida	Y	Y	Y	Y
Georgia	N	N	N	Y
Idaho	N	Y	N	N
Indiana	N	Y	N	N
Iowa	N	Y	N	N
Kentucky	Y	Y	Y	Y
Louisiana	Y	Y	Y	Y
Maryland	N	Y	N	N
Michigan	Y	Y	N	N
Minnesota	N	N	Y	Y
Mississippi	N	Y	N	N
Missouri	Y	Y	Y	N
Nebraska	N	Y	N	N
Nevada	N	Y	N	N
New Hampshire	Y	N	N	N
New Jersey	Y	Y	N	N
New Mexico	Y	N	N	N
Oklahoma	N	N	N	N
South Carolina	N	Y	N	N
Tennessee	N	N	Y	N
Texas	N	Y	Y	Y
Virginia	Y	Y	Y	N
Wyoming	Y	N	N	N

## Discussion

Overall, we found that more than half of states (59%) that currently have dyslexia legislation have some mention of language-related skills. Within these states, we found that language keywords were most commonly found in areas of legislation pertaining to intervention guidelines (66%), followed by guidelines for screening and assessing students (45%). A total of ten states also had legislation which included either specific or vague language in the description of disorders that fell under the scope of the legislation, including two states that specifically mention “language disorders.” These results indicate that a substantial portion of states have legislation that can potentially be interpreted to support the notion of the importance of screening and intervention for oral language skills in schools.

### “Vocabulary” and “comprehension” were the most frequently used language keywords

Of the language-related codes that we analyzed, the two most prevalent ones were “comprehension” and “vocabulary.” This result is not surprising, as both of these keywords are explicitly included in the IDA definition of dyslexia, which has been adopted by the majority of states with dyslexia legislation. We omitted instances of these keywords that appeared in sections of legislation which defined dyslexia. However, it is likely that states that use the IDA definition of dyslexia would include comprehension and vocabulary as screening and intervention targets, as they are indicated as being secondarily affected in children with dyslexia. Research has shown that children with dyslexia have deficits in word learning, vocabulary, and comprehension across the lifespan (Alt et al., 2017; Ransby & Swanson, 2003; Snowling et al., 2019).

Overall, codes that contained the word “language,” including *receptive language, expressive language, language development,* and *oral/spoken language*, appeared less frequently across dyslexia legislation. Of these, “oral/spoken language” was mentioned the most, in legislation from 12 states. This may reflect the common description of dyslexia as a “language-based learning disability,” with the phonological component of language typically receiving the most focus (Adlof & Hogan, 2018; Lyon et al., 2003).

### Short-term and long-term implications for advocacy

There are potential short-term implications of the inclusion of these language keywords in legislation from select states. In the thirteen states that mention language skills in their screening requirements, there is potential for schools to utilize existing frameworks for collecting student data on language ability to informally screen for oral language. For example, if a school already routinely assesses vocabulary skills in their dyslexia screener, these data can be used to identify children who may be at risk of a language disorder such as DLD. Vocabulary, word learning, and comprehension have all been consistently shown to be areas of deficit for many children with DLD (Hogan et al., 2014; Kan & Windsor, 2010). In schools that do not systematically screen for oral language skills more formally, this may be a way to utilize data that is already being collected as a temporary informal language screening tool.

We also identified ten states that include specific references to either “language disorder” or related disorders that can be interpreted to include DLD. In these states, there is a clearer pathway towards beginning to advocate for systematic language screening in schools using existing legislation. If it is included in current school screening guidelines to screen for dyslexia and related language disorders, then these schools may already be required to have some form of language screening in place. This provides an opportunity for educators and clinicians in schools to implement evidence-based language screeners more formally even without legislation that specifically mandates universal language screening.

However, the results of this study also indicate that most states do not have legislation that includes language disorders within the scope of their guidelines. This is an indication that additional legislation that addresses language disorders specifically is needed. Previous research has highlighted the need for universal language screening (Adlof & Hogan, 2019) and proposed potential policy changes to support progress towards universal language screening in schools. The parent advocates and grassroots movements for children with dyslexia have resulted in an explosion of dyslexia-related legislation in recent years (Gearin et al., 2018). The success of these efforts may be attributed in part to the compelling written narratives, submitted by stakeholders to legislative bodies, which often utilize a “conversion narrative” structure (Gabriel, 2020). Another factor that may explain why similar legislation does not yet exist for DLD is the visibility and consistency of terms used to describe children with unexplained language difficulties (Georgan et al., 2023; Leonard, 2020).

DLD advocacy has grown in recent years, with organizations such as DLD and Me, The DLD Project, and Raising Awareness of DLD (RADLD). For example, advocacy from the American Speech-Language-Hearing Association (ASHA) resulted in an official communication from the U.S. Office of Special Education and Rehabilitative Services (OSERS), stating that the term DLD would be encompassed by the definitions of disability laid out in the IDEA (Hogan et al., 2023). This clarification built on the one stated in the 2015 Dear Colleague letter that clarified the use of the term “dyslexia” for special education eligibility (Yudin, 2015). Already, there is evidence that DLD advocacy groups can build from the fruits of dyslexia advocacy. These groups and other stakeholders for children with language disorders are well-positioned to carry out advocacy efforts for children with DLD similar to what has occurred for children with dyslexia, and can potentially bring about the same results with respect to legislation regarding language screening, language intervention, and language training for pre-service and in-service professionals. Such legislation should be grounded in contemporary research and integrated with existing risk frameworks in order to avoid some of the current issues in the landscape of dyslexia legislation (Odegard et al., 2025).

## Conclusions

Ultimately, we found that language-related skills are incorporated in current dyslexia legislation in some capacity in over half of states. There is variability in how much state legislation addresses language skills and the contexts in which language skills are mentioned. We also identified a subset of states that potentially include DLD within the scope of their dyslexia legislation. These findings serve as a starting point for understanding potential avenues for advocating for universal oral language screening in schools.

## Footnotes

1 Since the publishing of this work, the IDA has updated its definition of dyslexia (IDA, 2002). The new definition can be accessed here: https://dyslexiaida.org/2025-dyslexia-definition-project/
